# Soil Carbon, Nitrogen, and Phosphorus Cycling Microbial Populations and Their Resistance to Global Change Depend on Soil C:N:P Stoichiometry

**DOI:** 10.1128/mSystems.00162-20

**Published:** 2020-06-30

**Authors:** Gongwen Luo, Chao Xue, Qianhong Jiang, Yan Xiao, Fengge Zhang, Shiwei Guo, Qirong Shen, Ning Ling

**Affiliations:** a Jiangsu Provincial Key Lab for Solid Organic Waste Utilization, Jiangsu Collaborative Innovation Center for Solid Organic Waste Resource Utilization, Nanjing Agricultural University, Nanjing, China; b College of Agro-Grassland Science, Nanjing Agricultural University, Nanjing, China; Pacific Northwest National Laboratory

**Keywords:** global change, ecosystem function, functional gene, biogeochemical process, C mineralization, nutrient transformation, resistance, stoichiometry

## Abstract

Maintaining stability of ecosystem functions in the face of global change calls for a better understanding regulatory factors of functionally specialized microbial groups and their population response to disturbance. In this study, we explored this issue by collecting soils from 54 managed ecosystems in China and conducting a microcosm experiment to link disturbance, elemental stoichiometry, and genetic resistance. Soil carbon:nitrogen:phosphorus (C:N:P) stoichiometry imparted a greater effect on the abundance of microbial groups associated with main C, N, and P biogeochemical processes in comparison with mean annual temperature and precipitation. Nitrogen cycling genes, including bacterial *amoA-b*, *nirS*, *narG*, and *norB*, exhibited the highest genetic resistance to N deposition. The *amoA-a* and *nosZ* genes exhibited the highest resistance to warming and drying-wetting cycles, respectively. Soil total C, N, and P contents and their ratios had a strong direct effect on the genetic resistance of microbial groups, which was dependent on mean annual temperature and precipitation. Specifically, soil C/P ratio was the main predictor of N cycling genetic resistance to N deposition. Soil total C and N contents and their ratios were the main predictors of P cycling genetic resistance to N deposition, warming, and drying-wetting. Overall, our work highlights the importance of soil stoichiometric balance for maintaining the ability of ecosystem functions to withstand global change.

**IMPORTANCE** To be effective in predicting future stability of soil functions in the context of various external disturbances, it is necessary to follow the effects of global change on functionally specialized microbes related to C and nutrient cycling. Our study represents an exploratory effort to couple the stoichiometric drivers to microbial populations related with main C, N, and P cycling and their resistances to global change. The abundance of microbial groups involved in cellulose, starch, and xylan degradation, nitrification, N fixation, denitrification, organic P mineralization, and inorganic P dissolution showed a high stoichiometry dependency. Resistance of these microbial populations to global change could be predicted by soil C:N:P stoichiometry. Our work highlights that stoichiometric balance in soil C and nutrients is instrumental in maintaining the stability and adaptability of ecosystem functions under global change.

## Abstract (precis)

To be effective in predicting future stability of soil functions in the context of various external disturbances, it is necessary to follow the effects of global change on functionally specialized microbes related to C and nutrient cycling. Our study represents an exploratory effort to couple the stoichiometric drivers to microbial populations related with main C, N, and P cycling and their resistances to global change. The abundance of microbial groups involved in cellulose, starch, and xylan degradation, nitrification, N fixation, denitrification, organic P mineralization, and inorganic P dissolution showed a high stoichiometry dependency. Resistance of these microbial populations to global change could be predicted by soil C:N:P stoichiometry. Our work highlights that stoichiometric balance in soil C and nutrients is instrumental in maintaining the stability and adaptability of ecosystem functions under global change.

## INTRODUCTION

Soil microorganisms contribute to multiple ecosystem functions, including litter decomposition, nutrient cycling, primary production and the regulation of greenhouse emissions (1, 2). The roles of functionally specialized groups of microbes that carry out ecosystem functions are of pivotal importance for carbon (C) and nutrient cycling in terrestrial ecosystems (2, 3). For example, nitrogen (N) transformations such as fixation of atmospheric N into plant available ammonium, nitrification of ammonium into N oxides, and denitrification of NO_3_^−^ into N_2_O and N_2_ are exclusively performed by diazotrophs, nitrifiers, and denitrifiers, respectively (4). When the ability of a species to perform its functions is hampered due to altered environmental conditions, other organisms may replace the lost functional component (2, 5). When there is species turnover across space or time due to environmental change, enhanced soil biodiversity can serve as a reservoir of specialized functional groups that complement each other, thus increasing overall ecosystem sustainability (5, 6). As environmental pressure continues, there may be a loss or suppression of key soil microbial groups (e.g., ammonia oxidizers, denitrifiers, and cellulolytic decomposers) that could result in an abrupt shift in multiple ecosystem functions. Therefore, functional microbes that carry out integral biogeochemical processes are necessary for proper ecosystem functions.

The growing human modification of terrestrial ecosystems has motivated ecologists to address how global change will impact the provisioning of multiple functions (7–9). Given the strong control that microbial communities exert over critical biogeochemical processes, there is a growing consensus that accurate predictions of future ecosystem stability require a more mechanistic understanding of the ability of functional populations to withstand disturbances (termed resistance) (2, 3, 10). Soil microbial functions are resilient after environmental change, though community composition is irreversibly altered (10, 11). When disturbances cease, nutrient biogeochemical cycling and C transformations depend solely on the remaining microbial populations. For example, organic matter decomposition, N mineralization, and nitrification appear to have a strong capacity to recover from drying and rewetting events (12–14). This resilience may be regulated by taxonomic groups that possess the functional capacity to synthesize enzymes that mitigate deleterious effects (15, 16). Exploring the effects of global change on specific microbial populations and their resistance is crucial in order to assess the stability and adaptability of ecosystem functions.

In the case of transient disturbances, numerous studies have reported stress-induced bursts of microbial C and N cycling. These tightly coupled processes may indeed be sensitive to projected climate change, such as warming and reduced precipitation (9, 17). Though soil microorganisms play a pivotal role in phosphorus (P) cycling through organic P mineralization and inorganic P dissolution, the impact of disturbances on microbially driven P fluxes has received much less attention than those of C and N (18). Overall, the main objectives of this study were to reveal the effects of mean annual temperature (MAT) and precipitation (MAP) and soil C:N:P stoichiometry on the microbial populations associated with the major pathways of C, N, and P cycling and to parallelly assess their integrated response to drying-wetting cycle, warming, and N deposition.

**FIG 1:**
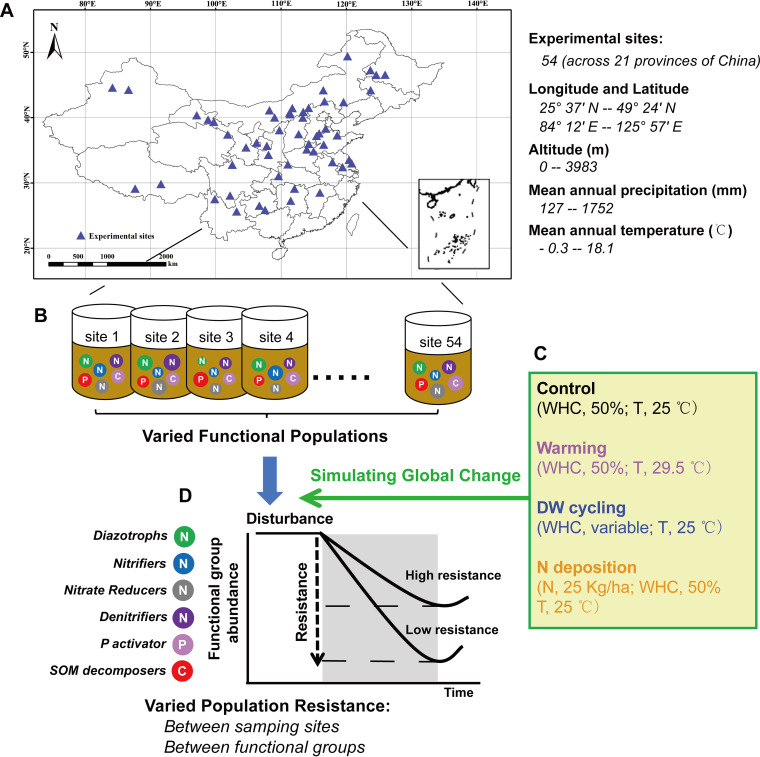
Fifty-four sampling sites were located in 21 provinces of China and spanned the major alfalfa growing region. These sites span a gradient of longitude and latitude and possess varied soil C:N:P stoichiometry (A). Considering the asymmetry in mean annual temperature and precipitation and soil C:N:P stoichiometry, the population patterns of C, N, and P cycling microbial groups may exist differently in the 54 sampling sites (B). Methodological framework explaining the design of microcosm experiments and the condition in all treatments (including the environmental control, drying-wetting cycles, warming, and N deposition treatments [C]). Varied population resistance of C, N, and P cycling groups may occur among sampling sites, and among functional genes (D). The letters C, N, and P in the circles represent different populations involved in the C, N, and P cycling processes, respectively. Diazotrophs regulate N fixation, nitrifiers regulate nitrification, nitrate reducers regulate nitrate reduction, denitrifiers regulate denitrification, P activators regulate organic P mineralization and inorganic P dissolution, and organic matter decomposers regulate cellulose, starch, and xylan degradation (Table 1). DW and WHC, drying-wetting and water holding capacity, respectively.

An increasing body of literature supports the idea that microbial communities exhibit spatial patterns at different scales (19). To more closely assess this pattern and to gain insight into the underlying functional processes and stability that are dependent on MAT and MAP and soil C:N:P stoichiometry (including soil total C, N, and P contents and their ratios), we used soils of 54 alfalfa planting systems from 21 provinces in China. Soil C:N:P stoichiometry constitutes an inherent link between biogeochemistry and the processes and structure within soil food webs (20). We hypothesized that the abundance of main C, N, and P cycling microbial groups would be mainly governed by soil C:N:P stoichiometry rather than MAT and MAP. Soil C and N biogeochemical cycling processes are sensitive to the projected climatic change (21, 22). Thus, we hypothesized that C and N cycling groups of alfalfa planting soils might be exhibited a higher resistance to N deposition than the drying-wetting cycles and warming. Microbial physiological feedback (such as regulating osmotic pressure) and functional recovery in response to stressors require a large investment of energy and resources (7, 23). Following this perspective, we further postulated that soil C:N:P stoichiometry might be a strong predictor of the population resistance of aforementioned functional groups. Soils were incubated subsequently for 1 month under different conditions simulating the expected impacts of changes in water availability (control versus drying-wetting cycles), temperature (control versus 4.5 °C warming), and N deposition (control versus 25 kg of N ha^−1 ^year^−1^) (Fig. 1). Quantitative PCR (qPCR) was employed to quantify the target genes associated with the biogeochemical cycling of C (cellulose, starch, and xylan degradation), N (nitrification, N fixation, and denitrification) and P (P mineralization and dissolution) (Table 1).

**TABLE 1:** The encoded protein and the functional process of each gene

Gene^*a*^	Encoded protein	Functional process	Reference
Diazotroph			
*nifH*	Nitrogenase (contains an Fe protein and a Mo-Fe protein)	Nitrogen fixation (N_2_ → NH_4_^+^)	74
Nitrifiers			
*amoA-a*	Ammonia monooxygenase	Nitrification (NH_4_^+^ → NH_2_OH)	4
*amoA-b*	Ammonia monooxygenase	Nitrification (NH_4_^+^ → NH_2_OH)	4
Nitrate reducer			
*narG*	Nitrate reductase (Mo containing)	Nitrate reduction (NO_3_^−^ → NO_2_^−^)	75
Denitrifiers			
*nirK*	Nitrite reductase (Cu containing)	Denitrification (NO_2_^−^ → NO)	76
*nirS*	Nitrite reductase (cytochrome *cd1*)	Denitrification (NO_2_^−^ → NO)	76
*norB*	Nitric oxide reductase	Denitrification (NO → N_2_O)	4
*nosZ*	Catalytic subunit of multi-Cu N_2_O reductase	Denitrification (N_2_O → N_2_)	77
P activators			
*phoD*	Alkaline phosphomonoesterase	Organic P mineralization	78
*phoC*	Acid phosphomonoesterase	Organic P mineralization	79
*BPP*	Phytase	Phytic acid mineralization	80
*pqqC*	Pyrroloquinoline-quinone synthase	Inorganic P dissolution	78
SOM decomposers			
*fungcbhIR*	Cellulolytic enzymes	Cellulose degradation	81
*GH74*	Endoglucanase/putative xyloglucan-specific endo-β-glucanase	Cellulose degradation	81
*GH31*	α-Glucosidase	Starch degradation	81
*GH51*	α-l-Arabinofuranosidase	Xylan side chain (arabinan) degradation	81

a *fungcbhIR*, fungal glycoside hydrolase family 7 cellobiohydrolase I genes; *GH31*, *GH51*, and *GH74*, glycoside hydrolase family 31, glycoside hydrolase family 51, and glycoside hydrolase family 74 genes, respectively; *amoA-a* and *amoA-b*, archaeal and bacterial ammonia monooxygenase gene (*amoA*), respectively. *BPP*, beta-propellerphytase.

## RESULTS

### Population abundance of functional microbial groups across ecosystem scales.

Quantitative PCR was used to quantify the abundance of the genes associated with cellulose degradation (*fungcbhIR* and *GH74* genes), starch degradation (*GH31*), xylan (or arabinan) degradation (*GH51*), nitrification (*amoA-a* and *amoA-b*), N fixation (*nifH*), and denitrification (*narG*, *nirK*, *nirS*, *nosZ*, and *norB* genes), organic P mineralization (*phoD*, *phoC*, and *BPP* genes), and inorganic P dissolution (*pqqC*) (Table 1). Inclusive of all sites, there was a positive relationship among the C, N, and P cycling microbial populations (*R*^2^ = 0.42 to ∼0.57; *P* < 0.001 [see Fig. S1 in the supplemental material]), and the significant relationships were observed among most of components of these populations (Fig. S2).

10.1128/mSystems.00162-20.1 FIG S1 The relationships between the C, N, and P cycling microbial populations. The blue fitted lines are from the regression. Only significant fitted lines are displayed on the graphs. Shaded areas show 95% confidence intervals of the fit. Download FIG S1, TIF file, 0.9 MB. Copyright © 2020 Luo et al. 2020 Luo et al. This content is distributed under the terms of the Creative Commons Attribution 4.0 International license.

10.1128/mSystems.00162-20.2 FIG S2 Correlations among the C, N, and P cycling gene abundances. The matrix was produced based on percent identity in R statistical software using the package corrgram. Asterisks indicate significance at *P* values of <0.05, <0.01, and <0.001 (*, **, and ***, respectively), and the white space indicates that there was no significance. Download FIG S2, TIF file, 1.4 MB. Copyright © 2020 Luo et al. 2020 Luo et al. This content is distributed under the terms of the Creative Commons Attribution 4.0 International license.

**FIG 2:**
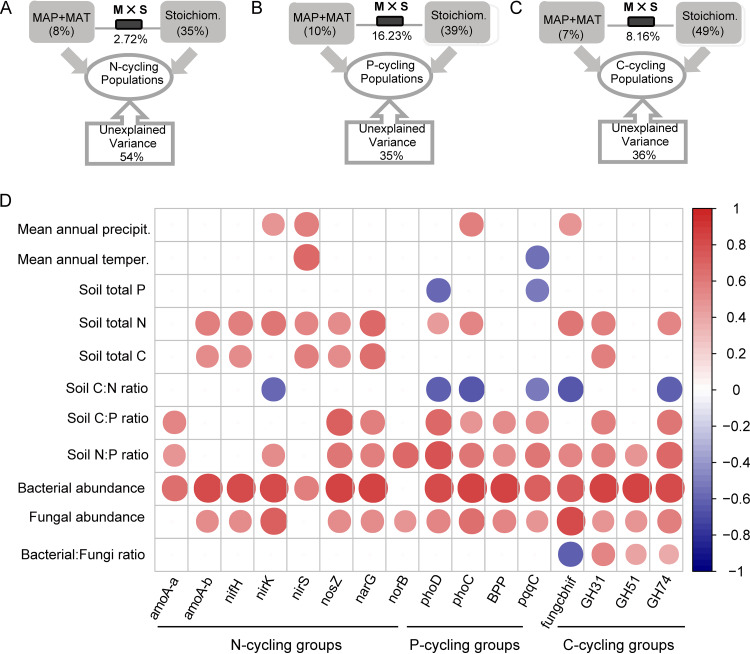
(A to C) Variance partitioning analysis map of the individual and combined explanatory power of mean annual temperature (MAT) and precipitation (MAP) and soil C:N:P stoichiometry on functional populations. Carbon cycling groups include the principal genes driving cellulose degradation (*fungcbhIR* and *GH74*), starch degradation (*GH31*), and xylan/arabinan degradation (*GH51*); N cycling groups include the genes driving nitrification (*amoA-a* and *amoA-b*), N fixation (*nifH*), and denitrification (*narG*, *nirK*, *nirS*, *nosZ*, and *norB*); and P cycling groups include the genes driving organic P mineralization (*phoD*, *phoC*, and *BPP*) and inorganic P dissolution (*pqqC*) (Table 1). Soil C:N:P stoichiometry includes soil total C, N, and P contents and their ratios. “M × S” stands for the variation explained jointly by MAT, MAP, and soil C:N:P stoichiometry. (D) The potential relationships of individual gene abundance with MAT, MAP, soil C:N:P stoichiometry, and bacterial and fungal abundance. The blue and red colors show, respectively, a negative and positive relationships between two variables. The deeper the color and the larger the circle, the stronger the relationships. The box without a circle indicates an absence of differences.

MAT and MAP and soil C:N:P stoichiometry (including soil total C, N, and P contents and their ratios) impacted the abundances of C, N, and P cycling genes (Fig. 2A to C). Soil C:N:P stoichiometry (35% to ∼49%; *P* < 0.001) had a greater impact on the abundances than did the MAT and MAP (7% to ∼10%; *P* < 0.01) based on variance partitioning analysis. The variation explained jointly by these variables was 16% and 8% of the total variation in P and C cycling groups, respectively (Fig. 2A to C; *P* < 0.01).

Heat map analysis of relational networks dissected the relationships between MAT and MAP and soil C:N:P stoichiometry against the abundance of C, N, and P cycling genes (Fig. 2D). Soil total N content, and soil C/P and N/P ratios, exhibited positive relationships with the majority of functional gene abundance (Fig. 2D; all *P* < 0.05). Soil total C content was positively related to the abundance of N cycling groups (including *amoA-b*, *nifH*, *narG*, *nirS*, and *nosZ* genes; all *P* < 0.05). Soil total P content was negatively correlated with the abundance of P activators (*phoD* and *pqqC* genes; all *P* < 0.05). Soil C/N ratio exhibited a negative relationship not only with the abundance of P activators (*phoD*, *phoC*, and *pqqC* genes) but also with denitrifiers (*nirK*) and cellulolytic decomposers (*fungcbhIR* and *GH74* genes) (Fig. 2D; all *P* < 0.05). MAT or MAP was found to have a significant relationship only with the abundance of *nirK*, *phoC*, *pqqC*, and *fungcbhIR* genes. Bacterial and fungal abundance exhibited positive relationships with the majority of functional gene abundance, while the ratio of bacterial to fungal abundance was related to the gene abundance within C cycling groups (*fungcbhIR*, *GH31*, *GH51*, and *GH74* genes) (Fig. 2D; all *P* < 0.05).

### Population resistance of functional microbial-groups to simulated global change.

**FIG 3:**
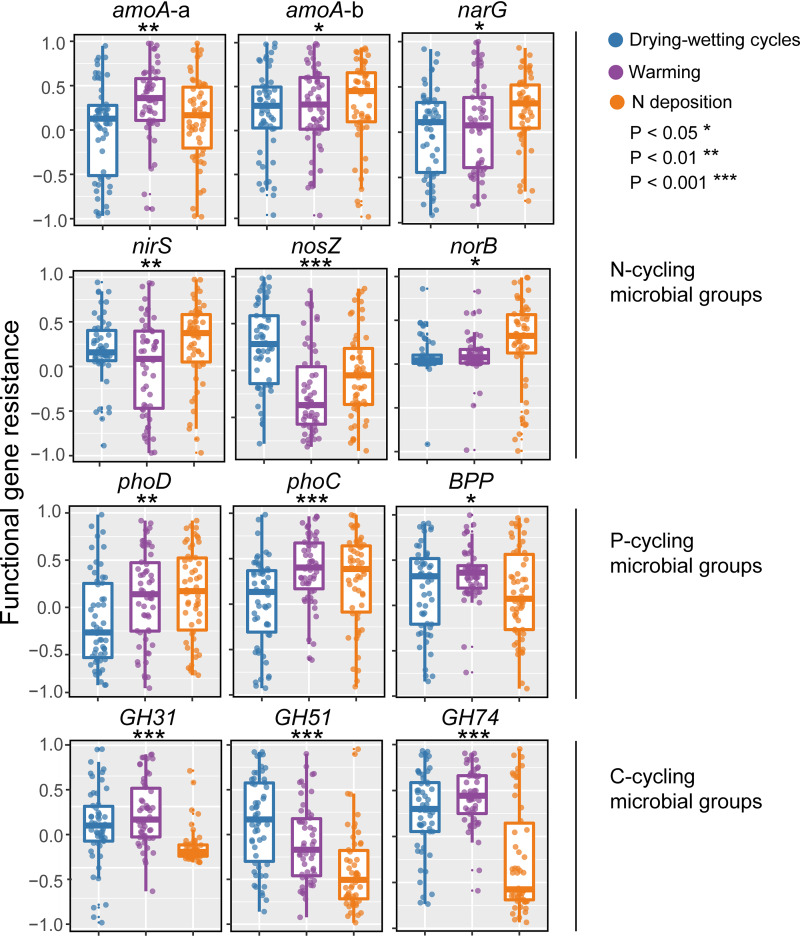
Effects of warming, drying-wetting cycles, and N deposition on the resistance of genes involved in C, N, and P cycling. Detailed information on functional genes is provided in Table 1. The upper and lower boundaries of each box indicate the 75th and 25th percentiles, respectively, and the midline marks the median of the distribution of the resistance values. The asterisks indicate that the genes showed a significant difference in the resistance to the three perturbations.

Among the biomarkers of C, N, and P cycling groups, the *phoD* gene presented the lowest resistance to drying-wetting cycles, the *nosZ* and *GH51* genes exhibited the lowest resistance to warming, and the *GH51* and *GH74* genes were least resistant to N deposition (Fig. S3). Nitrogen cycling genes, including *amoA-b*, *nirS*, *narG*, and *norB*, exhibited the highest genetic resistance values to N deposition, and the *amoA-a* and *nosZ* genes exhibited the highest resistance to warming and drying-wetting cycles, respectively (Fig. 3; all *P* < 0.05). The genetic resistance of P cycling genes (*phoD* and *phoC*) illustrated an absence of differences between warming and N deposition, while the resistance to the two stressors was higher than that to drying-wetting cycles and the resistance of *BPP* gene to warming and drying-wetting cycles was higher than that to N deposition (Fig. 3; all *P* < 0.05). Carbon cycling genes, including *GH31*, *GH51*, and *GH74*, exhibited lower genetic resistance to N deposition than to the drying-wetting cycles and warming (Fig. 3; all *P* < 0.05). Inclusive of all sites, there was a positive relationship among the C, N, and P cycling microbial populations under the three stressors (*R*^2^ = 0.33 to ∼0.62; *P* < 0.001 [Fig. S4]).

10.1128/mSystems.00162-20.3 FIG S3 Resistance of genes involved in C, N, and P cycling to drying-wetting cycles (A), warming (B), and N deposition (C). The following genes were assessed: *fungcbhIR* and *GH74* as the biomarkers of cellulose degradation, *GH31* as the biomarker of starch degradation, *GH51* as the biomarker of xylan/arabinan degradation, *amoA-a* and *amoA-b* as the biomarkers of nitrification, *nifH* as the biomarker of N fixation, *narG*, *nirK*, *nirS*, *nosZ*, and *norB* as the biomarkers of denitrification, *phoD* and *phoC* as the biomarkers of organic P mineralization, *BPP*, as the biomarker of phytic acid mineralization, and *pqqC* as the biomarker of inorganic P dissolution (Table 1). Download FIG S3, TIF file, 1.7 MB. Copyright © 2020 Luo et al. 2020 Luo et al. This content is distributed under the terms of the Creative Commons Attribution 4.0 International license.

10.1128/mSystems.00162-20.4 FIG S4 The relationships between the C, N, and P cycling microbial populations after the simulated disturbance of drying-wetting cycle, warming, and N deposition. The blue fitted lines are from the regression. Only significant fitted lines are displayed on the graphs. Shaded areas show 95% confidence intervals of the fit. Download FIG S4, TIF file, 2.5 MB. Copyright © 2020 Luo et al. 2020 Luo et al. This content is distributed under the terms of the Creative Commons Attribution 4.0 International license.

### Regulatory pathway of multiple drivers on functional population resistances.

**FIG 4:**
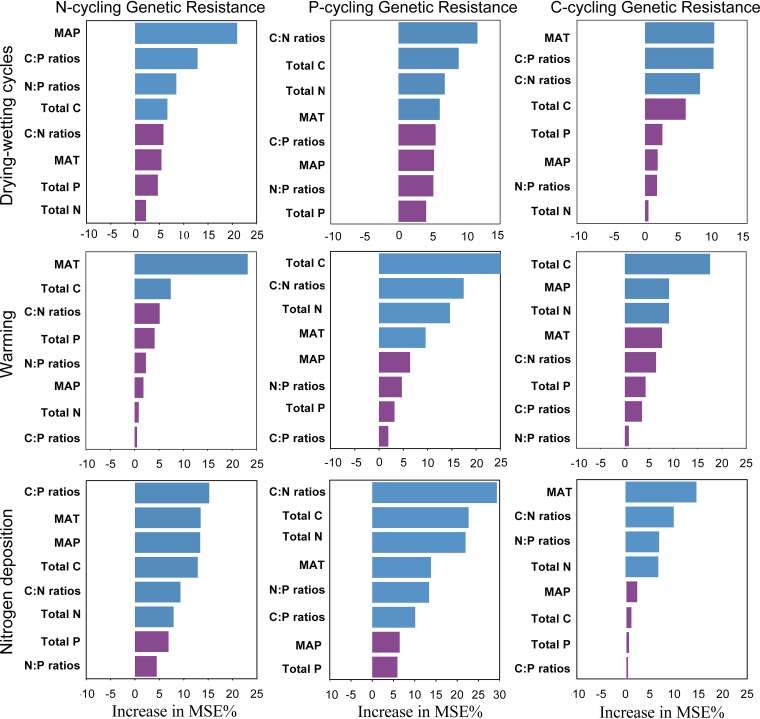
Random forest mean predictor importance (percentage of increase of mean square error [MSE]) of MAT and MAP and soil C:N:P stoichiometry as the drivers of C, N, and P cycling genetic resistance to warming, drying-wetting cycles, and N deposition. This accuracy importance was computed for each tree and averaged over the forest (500 trees). Blue fill indicates significance level at a *P* value of <0.05.

Mean annual precipitation exhibited the highest mean predictor importance (MPI; 21%) in the explanation of N cycling genetic resistance to drying-wetting cycles (random forest models [Fig. 4]). Mean annual temperature exhibited the highest MPI (23%) in the explanation of N cycling genetic resistance to warming. Soil C/P ratio exhibited the highest MPI (15%) in the explanation of N cycling genetic resistance to N deposition, followed by the MAT and MAP (Fig. 4). Soil total C and N contents and their ratios were selected by random forest analyses as the main predictors of the P cycling genetic resistance to the three stressors. Soil C/P ratio (10%) and MAT (10%) exhibited the highest MPI in the explanation of C cycling genetic resistance to drying-wetting cycles, and parallelly soil total C (18%) and MAT (15%) exhibited the highest MPI in the explanation of C cycling genetic resistance to warming and N deposition, respectively (Fig. 4).

**FIG 5:**
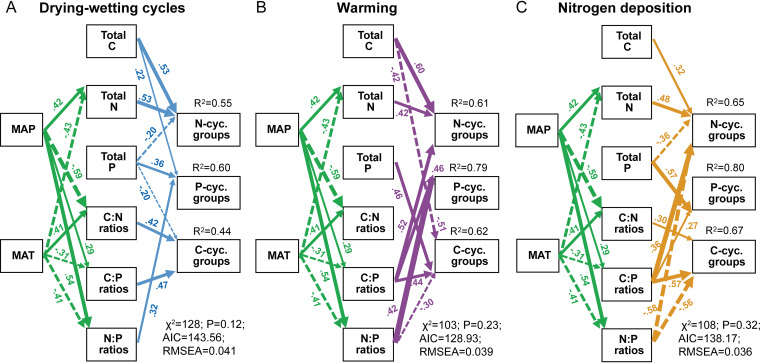
Structural equation modeling describing the effects of MAT and MAP and soil C:N:P stoichiometry on the population resistance of C, N, and P cycling microbial groups to drying-wetting cycles (A), warming (B), and N deposition (C). Solid and dashed arrows indicate the positive and negative effects, respectively. Numbers adjacent to arrows are indicative of the effect size of the relationship (termed standardized path coefficients). For simplicity, only significant direct effects were plotted (*P* < 0.05). *R*^2^ indicates the proportion of variance explained.

Assessment via structural equation modeling further provided mathematical evidence for the regulatory effect of the MAT and MAP and soil C:N:P stoichiometry on genetic resistance of the N, P, and C cycling microbial groups (Fig. 5). Both the MAT and MAP could directly affect soil C:N:P stoichiometry, which resulted in the ultimate regulation of genetic resistance (Fig. 5). For example, the MAP directly affected soil total N content and this regulatory effect mediated N cycling genetic resistance to climate change and N deposition. Soil C, N, and P contents and their ratios had a stronger direct effect on the genetic resistance of microbial groups. These drivers exhibited varied effects coefficient and direction among the three stressors (Fig. 5). For example, soil total C and N contents exhibited a consistently positive effect on the genetic resistance of N cycling groups, while the soil C/P ratio had a consistently positive effect on the genetic resistance of C, N, and P cycling microbial groups.

## DISCUSSION

### Effects of soil C:N:P stoichiometry on functionally specialized microbial populations.

In agreement with our hypothesis, the abundance of main functional genes involved in the biogeochemical cycling of C (cellulose, starch, and xylan degradation), N (nitrification, N fixation, and denitrification), and P (organic P mineralization and inorganic P dissolution) was mainly governed by soil C:N:P stoichiometry rather than MAT and MAP (Fig. 2). This is consistent with previous work showing that multiple edaphic factors, including soil physicochemical properties, temperature, and moisture, either together or individually influence microbially driven processes (19, 24, 25).

This study provided the opportunity to reveal linkages that impact biologically mediated N transformation processes of alfalfa planting soils. For example, the abundance of diazotrophs (*nifH* gene) exhibited a positive relationship with that of nitrifiers, nitrate reducers, and denitrifiers (Fig. S2). In this ecosystem, inputs of N to soil are generally dependent on biological N fixation (4). The sequences of *nosZ* and *nirS* are amplified clustered with the N-fixing bacteria *Azospirillum* and Bradyrhizobium japonicum, which demonstrates a potential relationship between biological N fixation and denitrification (26). The subsequent production of ammonium (mineralization), conversion of ammonium to nitrate (nitrification), and loss of nitrate via denitrification should exhibit a dependence on the prerequisite process (4, 25). Thus, a broader appreciation of N biogeochemical cycling in ecosystems can be attained synthetically via simultaneous analyses of the populations of diazotrophs, nitrifiers, nitrate reducers, and denitrifiers. The C, N, and P requirements of microbial biomass differ among species and are not homeostatic through time (27, 28). As expected, the majority of N cycling gene abundances were related to soil C and N contents and to C/P and N/P ratios (Fig. 2). For example, the abundance of the *nifH* gene was correlated with soil total C and N contents because fixed N can drive interlinked N and C cycling events, including mycorrhizal symbiosis and litter decomposition (29), or N fixation in alfalfa planting soils can add to C availability. This finding suggests that resource allocation processes bring C and N acquisition into stoichiometric balance.

Soil P biochemical processes are tightly coupled to C and N cycling (30, 31), which supports the positive relationships between the functional populations involved in C, N, and P transformations (Fig. 2 and Fig. S1). For example, *Bradyrhizobium*, a free-living and symbiotic diazotroph, plays an important role in coupling soil N and P cycling. To respond to P stress, some *Alphaproteobacteria* (e.g., *Bradyrhizobium* species) sacrifice N in order to obtain more available P by altering expression of P cycling genes (such as *phoD* and *phoC*) or the catalytic efficiency of phosphatases (32–34). Soil total P content was negatively correlated with the abundance of the *phoD* and *pqqC* genes, which facilitate P availability by encoding exoenzymes (Fig. 2). This result was consistent with our previous studies (18). Soil phosphatase activity and *phoD* abundance have been found to be linearly negatively correlated with available P concentration (18, 35). Under high-P conditions, it is likely that the control of microbial population is driven not by P availability but by the need for C (36–38). Microbial phosphatases and other enzymes are also involved in organic C mineralization in order to promote the availability of P and C from organophosphorylated compounds (36, 37). Thus, P transformation is inherently coupled, to a degree, with C mineralization (Fig. S1 and S2). The high soil C/N ratio indicates efficient degradation of fresh plant matter, which is important for the subsequent C sequestration (39). Soil C/P and N/P ratios have been demonstrated as the main predictors of the abundance and diversity of the *phoC*- and *phoD*-harboring microbial populations. Therefore, the abundance of P cycling genes was correlated with soil total C, N, and P contents (Fig. 2). Gaining a deeper understanding of the environmental and resource dependency of functional populations and their ability to withstand disturbances can be used to predict the ecosystem service sustainability.

### Stability of soil microbial functions to simulated global change.

The abundances of genes involved in soil C, N, and P cycling were differentially resistant to stress induced by simulated global change (Fig. 3 and Fig. S3). The mechanisms responsible for these idiosyncrasies likely result from the various diversities, metabolic versatilities, and environmental tolerances of specific functional groups (40, 41) and reflect complex feedback loops within the soil-microorganism system. For example, both bacterial and fungal isolates are known to induce survival strategies against desiccation and rewetting, including the accumulation of compatible solutes studied for *Streptomycetes* (23), exopolysaccharide production studied in *Pseudomonas* and *Acidobacteria* (42), and the induction of dormant phases such as through spore production (16). Therefore, indigenous functional groups may select for characteristic patterns in responding to global change by implementing varied physiological strategies that render them tolerant across dynamic water potentials.

Due to the ecophysiological diversity within functional microbial groups (8, 9), it is unlikely that all members of a group share similar ecophysiological characteristics. Thus, they are unlikely to react to different disturbances in the same manner. The response of functional groups to drying-wetting cycles, warming, and N deposition, therefore, followed different genetic resistances (Fig. 3). The sensitivity of soil N cycling to climate change is well accepted, as it has been declared that comparably small changes of temperature or moisture regimens affect nitrification and gross or net N mineralization (43–45). Drought or reduced soil moisture generally decreases N mineralization rates (46, 47). As assumed, N cycling genes, including *amoA-b*, *nirS*, *narG*, and *norB*, exhibited a higher genetic resistance to N deposition than to the drying-wetting cycles and warming (Fig. 3). Nitrogen deposition elevated spatial heterogeneity of soil microbial communities (48, 49). Heterogeneity might enable those functional populations to cope better with an uncertain future, and cells can switch stochastically between the different expression states under this circumstance (50).

Carbon cycling genes, including *GH31*, *GH51*, and *GH74*, exhibited a lower genetic resistance to N deposition than to the drying-wetting cycles and warming (Fig. 3). The microbial species involved in organic matter decomposition primarily belong to the fungus groups (51) which are generally more drought resilient than other species and continue to increase in biomass under water stress conditions (52). Nitrogen addition can enhance microbial C limitation, which may be attributed to an increase in recalcitrant organic matter through condensation reactions between mineral N and organic matter (53). Thus, there was a relatively lower resistance to N deposition of genes involved in cellulose degradation (*GH74*), starch degradation (*GH31*), and xylan degradation (*GH51*) than to drying-wetting cycles and warming (Fig. 3). This finding implies a lower phenotypic plasticity of those functional populations in resisting N deposition. Both soil organic and inorganic P may be solubilized following drying and rewetting events, which often induces not only a substantial pulse in soil respiration but also a leaching of dissoluble P (54). To meet their needs for available P, microorganisms are expected to obtain more P by the release of hydrolytic phosphatase (mineralization) and organic anions (solubilization). This mechanical theory suggests that phosphatase-harboring populations (*phoD* and *phoC* genes) are especially susceptible to drying-wetting cycles, with a relatively lower genetic resistance than to N deposition and warming (Fig. 3). According to these findings, to be effective in detecting or predicting potential changes in soil functional processes in the context of various global changes, it is necessary to focus on the specific environmental drivers that impact particular microbial populations of interest.

### Regulatory pathways of functional population resistance to global change.

Microorganisms frequently experiencing environmental changes have been found to be more resistant to such disturbances than populations with less exposure to the disturbances. For example, intensified exposure to rainfall (i.e., larger rainfall scenes separated by longer dry cycles) increases the drying-rewetting tolerance of soil bacteria (55). This perspective supports the notion that the MAT and MAP were consistently identified as the principal drivers of genetic resistance to drying-wetting cycles (Fig. 4 and 5). It is unclear how global warming will affect N fixation, with some predicting that future arctic environments will experience increased N fixation because of heightened enzyme activities and increased CO_2_ concentration (56). Conversely, N fixation may be inhibited by increased available N due to increased mineralization acting as a negative feedback upon this process (57). These contradictory results may be caused by different local climates, as our results showed that the MAT was consistently a primary predictor of N cycling genetic resistance to simulated warming (Fig. 4 and 5). This finding extends the understanding that past climatic conditions and disturbance history leave their imprint on the composition and function of microbial communities.

The production of enzymes is nutritionally and energetically expensive, requiring microbial investment of C (energy) and nutrients (termed cells’ economic policy) (9, 58). This may be overridden when organisms acclimate to stress by altering their survival strategies, such as the dependence on the microbial selection of substrates with various soil C:N:P stoichiometry (59, 60). Soil total C, N, and P contents and their ratios had a strong direct effect on the genetic resistance of microbial groups, which were dependent on the MAT and MAP, based on both the random forest and structural equation models (Fig. 4 and 5). Despite the soil stoichiometry being impacted by the fertilization management in agroecosystems, most studies conducted across ecosystems have reported that the variation in ecological stoichiometry is driven by the latitude, MAT, and MAP (20, 61, 62). Based on the components of previous discussion, phosphatase activity is highly responsive to changes in C, N, and P availability, which represents an important strategy by which organisms might be able to adjust to changes in biogeochemical cycles. These interactive effects may explain why the present study identified soil C and N contents and C/N ratio as strong predictors of resistance within P cycling populations (Fig. 4 and 5). With a sufficient C supply, increasing N availability meets the microbial C:N stoichiometric requirement and thus stimulates microbial activity, thereby accelerating C mineralization (27, 63). Nutrient limitation of soils may lead to a shift in the microbes from r- to K-strategists, the latter of which are considered able to decompose more stable organic matter for organic N or P acquisition and thus induce a higher activity of decomposition (64, 65). These findings may explain why the present study identified that the soil C/P, N/P, and C/N ratios were strong predictors and drivers of stress resistance of functional populations (Fig. 4 and 5).

Our study provides novel evidence that the populations of microbial groups involved in C, N, and P cycling were mainly governed by soil C:N:P stoichiometry rather than MAT and MAP. The response of functional groups to global change followed varied patterns, with the majority of functional genes exhibiting differential resistance to the same external stressor. Soil C:N:P stoichiometry had a strong direct effect on the genetic resistance of microbial groups, which were dependent on the MAT and MAP. Therefore, it is of vital importance to understand how C:N:P stoichiometry mediates the ability of microbial functions to withstand global change.

## MATERIALS AND METHODS

### Research site and soil sampling.

Soils were collected from 54 major alfalfa cultivation systems that are located in 21 provinces of China and span a gradient of precipitation and temperature (Fig. 1A). The interplay of environmental factors and local management techniques influences the consequences of soil properties across sampling sites. There are three primary reasons for the use of alfalfa planting systems in the present study: (i) alfalfa is a widely distributed perennial plant in China, which makes it beneficial for large-scale and multisample research; (ii) soil C, N, and P pools in most systems are usually complicated by plant residues with multiple stoichiometric ratios, but long-term alfalfa cropping reduces the effects of aboveground components or other vegetation on soil substrates; and (iii) alfalfa-driven N fixation promotes the formation of specific stoichiometric gradients of soil substrates. Lastly, these planting systems experience lower artificial disturbance than do farmland systems. Three plots at each site were randomly selected, from which five soil cores per plot were taken at a depth of 0 to 15 cm and combined. These three plot-level samples were sieved through 2.0-mm mesh to remove plant debris and rocks and then mixed thoroughly on a per-site basis to generate the final composite soil samples.

Soil total C and N contents were determined by the dry combustion of ground samples (100 mesh) in an elemental analyzer (vario MAX; Elementar, Germany). Soil total P content was determined by digestion with HF-HClO_4_ and, ultimately, determined via molybdenum blue colorimetry (66).

### Experimental design: soil incubation.

Soils for the microcosm experiment were incubated to evaluate the effects of simulated drying-wetting cycles, warming, and N fertilization on the resistance of functional microbial groups. In parallel, 5 g of soil from each site was loaded into ventilated canning jars, one for each global driver plus an environmental control. The environmental control was incubated at 25°C, the average land surface temperature for all sites (https://neo.sci.gsfc.nasa.gov/), at a 50% water holding capacity (WHC), the optimum water content for microbial respiration in soils with similar textures (67). The warming treatment had water conditions similar to those of the control but at an increased temperature (+4.5°C [Fig. 1C]). This temperature increase mimics global warming forecasts for the end of this century (17). The drying-wetting treatment was incubated at the same temperature as the control but included four cycles. Each cycle involved a 2-day wetting phase (a 50% soil WHC was achieved) and subsequent natural drying for 5 days (Fig. 1C). The N deposition treatment included the same temperature and water conditions as the control plus the equivalent of 25 kg of N ha^−1 ^year^−1^, which was added in the form of NH_4_NO_3_ during the first watering (Fig. 1C). This amount was selected based on the evaluation of N deposition in China (68). Moisture content was adjusted and maintained at a 50% WHC throughout the experiment for all treatments other than the drying-wetting treatment. A total of 216 samples (54 sites × 4 treatments) were incubated under the four treatments for a month.

### Quantifying functional groups.

Total genomic soil DNA was extracted using a Power Soil DNA isolation kit (Qiagen, Hilden, Germany) according to the manufacturer’s protocol. The DNA extracts were purified using a Wizard DNA cleanup system (Axygen Bio, USA), as recommended by the manufacturer. The DNA was then stored at –20°C for subsequent analyses.

Potential microbial functions were assessed by quantitative PCR (qPCR) of the key genes involved in cellulose degradation, starch degradation, xylan degradation, nitrification, N fixation, denitrification, organic P mineralization, and inorganic P dissolution (Table 1). Biomarkers of the bacterial 16S rRNA and fungal internal transcribed spacer (ITS) rRNA genes were quantified to calculate the ratio of fungal and bacterial abundances. Primer sequences for the target genes were adapted for qPCR, based on previous studies (Table S1). After running a serial dilution on the samples to test for PCR inhibition, each gene assay was performed with each sample run in triplicate. Standard curves were obtained using serial dilutions of a known amount of linearized plasmid DNA containing specific gene fragments. Quantification was performed with an ABI 7500 Cycle real-time PCR system (Applied Biosystems, Germany) in a 25-μl reaction mixture that included 12.5 μl of SYBR Premix *Ex Taq* (2×) (Tli RnaseH Plus), 0.5 μl of ROX reference dye II (50×) (TaKaRa Bio Inc., Japan), 0.5 μl of each primer (10 μM; forward primer and reverse primer), 1 μl of template, and 10 μl of double-distilled water (ddH_2_O) to bring the final volume up to 25 μl. Our method resulted in slightly different amplification efficiencies for these targeted genes, with *R*^2^ values between 0.94 and 0.98 (Table S1). These data were used to correct the gene abundance data before statistical analyses were performed.

10.1128/mSystems.00162-20.6 TABLE S1 Primers used for quantitative PCR and corresponding amplification cycling conditions. Download Table S1, DOCX file, 0.03 MB. Copyright © 2020 Luo et al. 2020 Luo et al. This content is distributed under the terms of the Creative Commons Attribution 4.0 International license.

### Decoupling genetic resistance.

Resistance (RS) was calculated as described by Orwin and Wardle (69):

(1) $$\text{RS}=1-\left(\frac{2|D_{0}|}{C_{0}+|D_{0}|}\right)$$

In this equation, *D*_0_ is the difference in the value of the response variables between the constant-condition samples (*C*_0_) and the treated samples at the end of the incubation period. This RS index increases monotonically with resistance, considers only the absolute differences between controlled and disturbed soils, and is standardized by the control value to enable valid comparisons between soils. It is bounded at +1 and −1, where a value of +1 indicates complete resistance (i.e., the disturbance did not cause any change in the response variable), a value of 0 indicates that there was a 100% change in the response variable compared to the control soil, and a value of −1 indicates that there was a change of more than 100% in the response variable compared to the control soil. We calculated the resistance of each gene abundance independently for each global change driver.

### Statistical analysis.

We used following equation to normalize the gene abundance belonging to the same functional group (70):

(2) $$x'=[\sum_{n=1}^{n}(\mathrm{xi}/\sum_{i=1}^{\text{i}}\mathrm{xi})]/n(i=1,2,3\ldots ;n=1,2,3\ldots )$$

where *x_i_* is the individual gene abundance of the samples, *i* and *n* indicate the numbers of samples and genes studied, respectively, and *x′* is the normalized abundance of the C, N, or P cycling microbial groups.

The statistical analyses were performed by using the IMB SPSS statistical software package version 20 (IBM Corporation, New York, NY) or R (version 3.1.1). Variance partitioning analysis with Hellinger-transformed data was used to determine the individual and combined variation explained by MAT and MAP and soil C:N:P stoichiometry on gene abundance. Soil C:N:P stoichiometry includes soil total C, N, and P contents and their ratios. A heat map was generated using the corrplot package to show the relationships of gene abundance with MAT, MAP, and soil C:N:P stoichiometry.

Classification random forest analysis proposed by Breiman (71), as described by Delgado-Baquerizo et al. (72), was employed to identify the most important and credible predictors of functional population resistance to global change within the components of soil C:N:P stoichiometry, MAT, and MAP. Random forest algorithms add an additional layer of randomness to bagging, and each tree used a different bootstrap sample of the data. The randomForest package provides an R interface to the Fortran programs implemented by Breiman and Cutler (http://www.stat.berkeley.edu/users/breiman/). The rfPermute package was used to estimate the significance of importance metrics for a random forest model by permuting the response variable. Random forest mean predictor importance (MPI) (percent increase in mean square error [MSE]) is used to characterize the main predictors.

Structural equation modeling (SEM) (73) was constructed to further reveal the indirect and direct effects of MAT, MAP, and soil C:N:P stoichiometry on the population resistance of functional groups to global change with a multivariate approach using AMOS software (IBM SPSS AMOS 20.0.0). Before such modeling, all data of model variables must be normalized. The first step in SEM requires establishing an *a priori* model based on the known effects and relationships among the stress resistance of functional populations, MAT, MAP, and soil C:N:P stoichiometry (Fig. S5). Variables included preselected major significant predictors of population resistance of functional groups from random forest analyses described above. The following metrics were used to evaluate the goodness of fit of our model: the root mean squared error of approximation (RMSEA) (the model has a good fit when the RMSEA is low [<0.05]), the chi-square value (χ^2^; the model has a good fit when the χ^2^ value is low), Fisher’s P statistic (the model has a good fit when 0.05 < *P* ≤ 1.00), and the Akaike information criterion (AIC) (the model has a good fit when the AIC is low). The different goodness-of-fit metrics used indicated that our *a priori* model considering the direct paths between MAT/MAP and the resistance of functional populations was not fitted to our data. Additionally, this *a priori* model indicates that there were no significant paths between MAT/MAP and resistance. Therefore, we constructed an optimal model that does not consider the direct (but indirect) effects of MAT and MAP on the resistance of functional populations (the paths between them were deleted in the optimal model). Ultimately, the resulting model was obtained by gradually modifying and optimizing the *a priori* model, which met the condition of goodness-of-fit metrics and confirmed the significance of paths.

10.1128/mSystems.00162-20.5 FIG S5 *A priori* structural equation model including direct and indirect effects of MAT, MAP, and soil C:N:P stoichiometry on the genetic resistance of microbial groups to N deposition, warming, and drying-wetting cycles. Download FIG S5, TIF file, 1.1 MB. Copyright © 2020 Luo et al. 2020 Luo et al. This content is distributed under the terms of the Creative Commons Attribution 4.0 International license.

### Data availability.

Our data set is publicly available through the Dryad database at https://doi.org/10.5061/dryad.05qfttf0d.
