# Supplementary figures and images for: Soil Carbon, Nitrogen, and Phosphorus Cycling Microbial Populations and Their Resistance to Global Change Depend on Soil C:N:P Stoichiometry
Source: mSystems. 2020 Jun 30;5(3):e00162-20. doi: 10.1128/mSystems.00162-20 (PMC7329320; doi:10.1128/mSystems.00162-20)

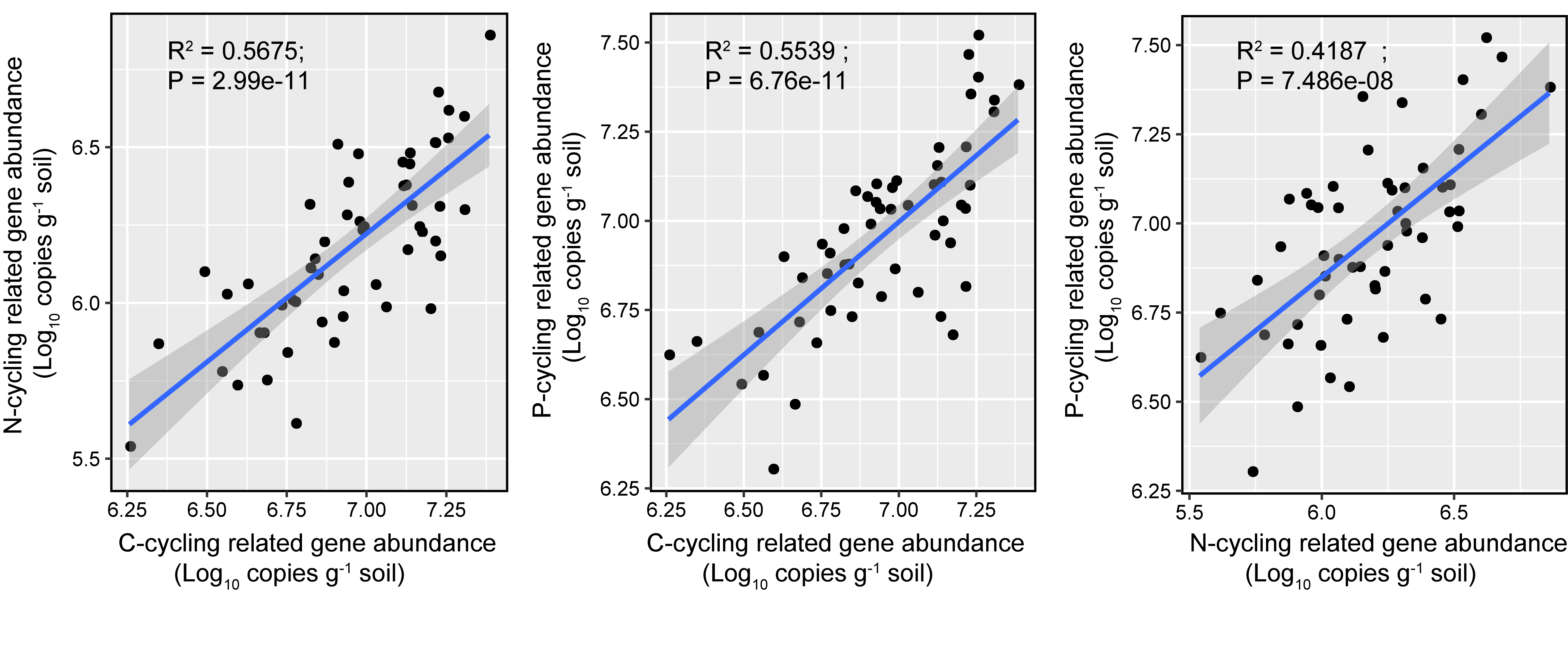

Supplement: FIG S1 [file mSystems.00162-20-sf001.tif]

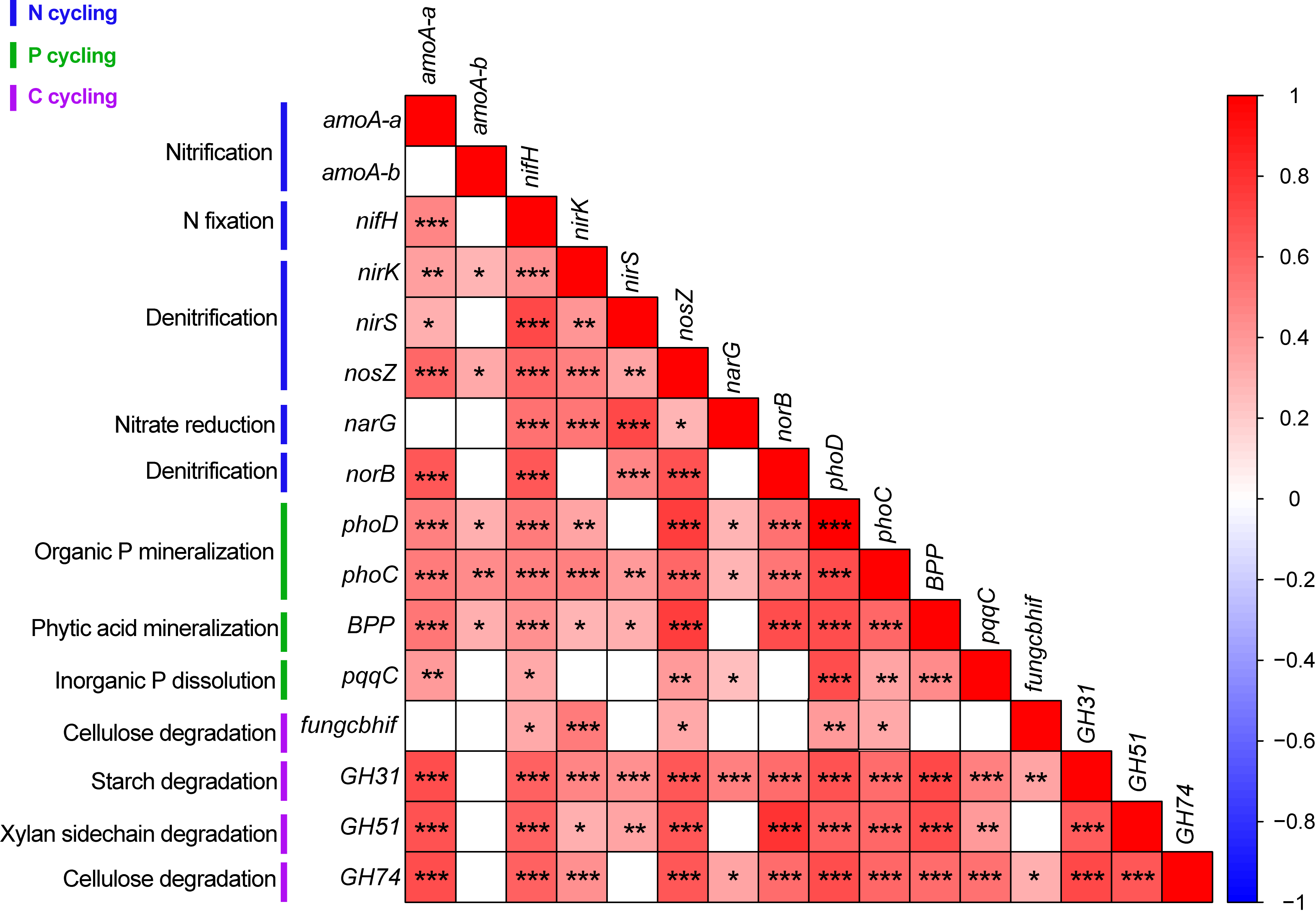

Supplement: FIG S2 [file mSystems.00162-20-sf002.tif]

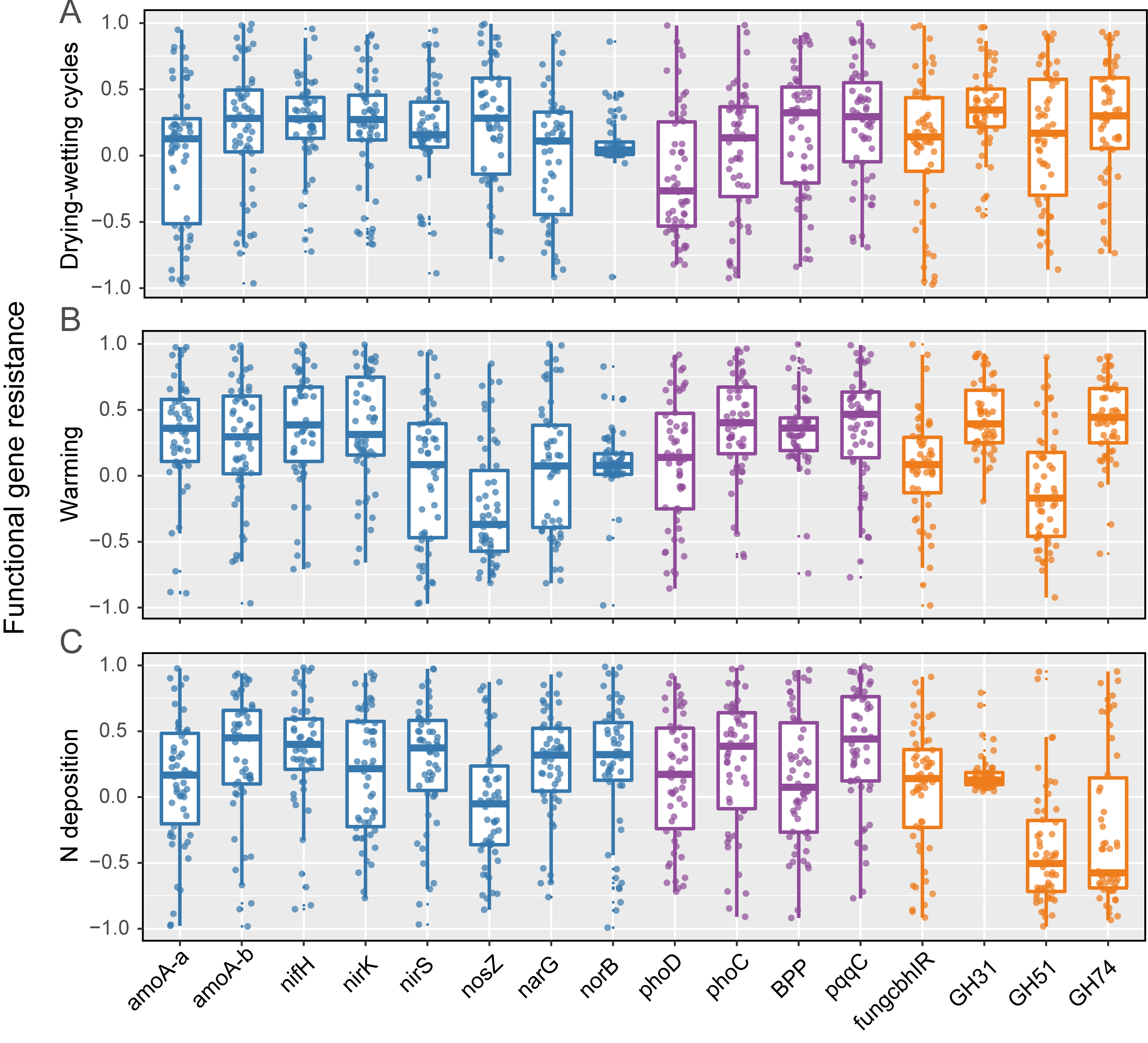

Supplement: FIG S3 [file mSystems.00162-20-sf003.tif]

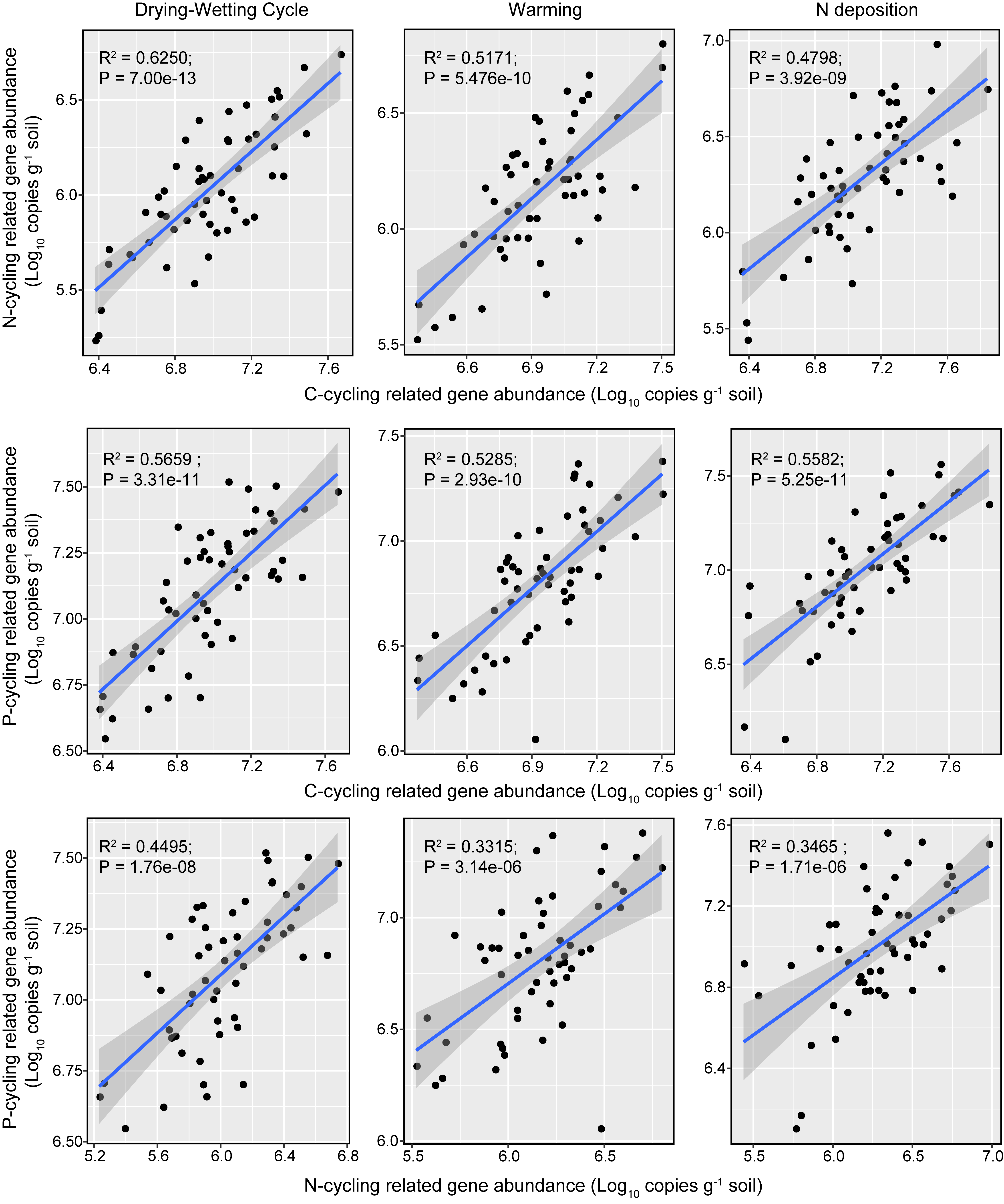

Supplement: FIG S4 [file mSystems.00162-20-sf004.tif]

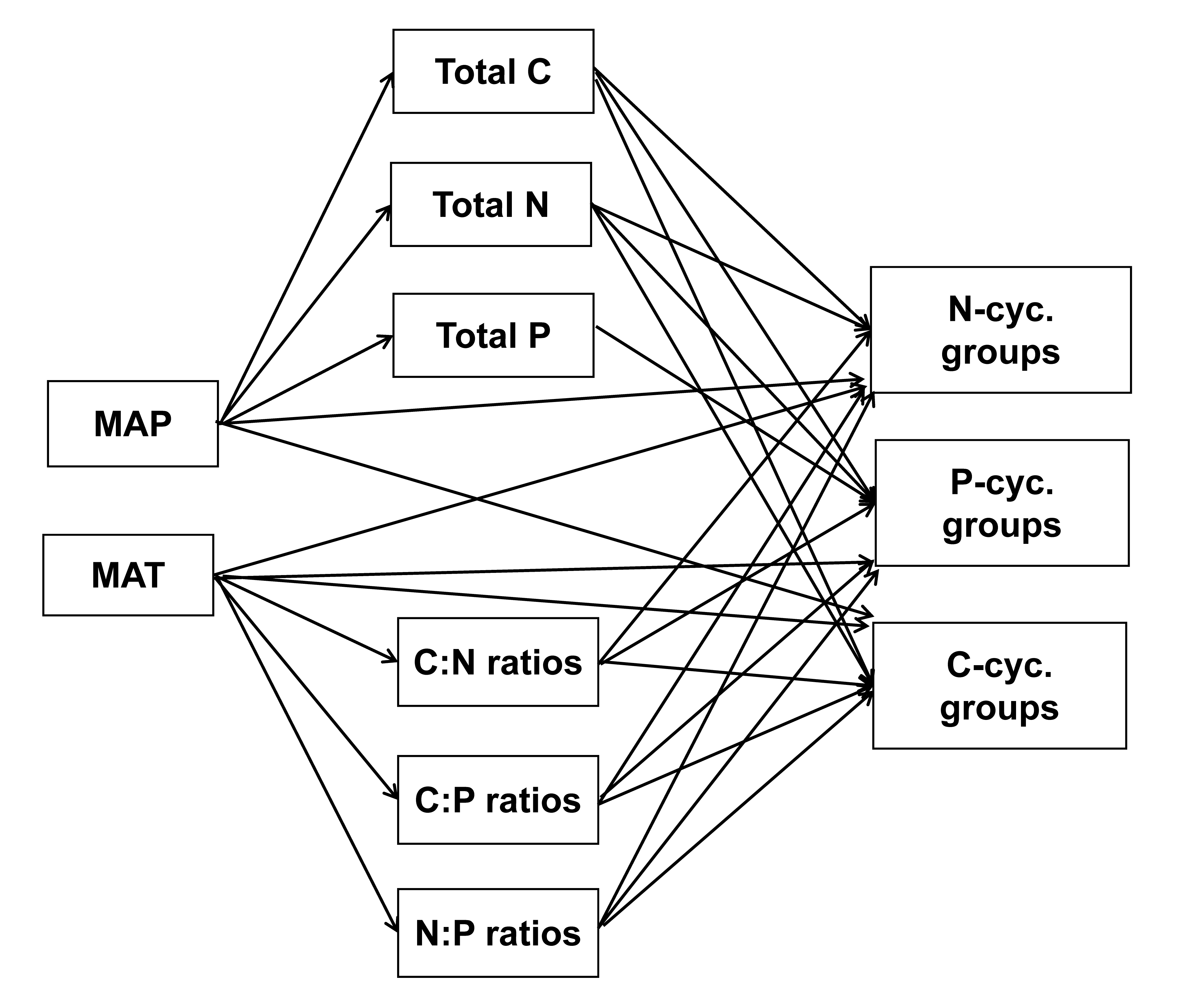

Supplement: FIG S5 [file mSystems.00162-20-sf005.tif]
