# Supplementary material for: Soil Carbon, Nitrogen, and Phosphorus Cycling Microbial Populations and Their Resistance to Global Change Depend on Soil C:N:P Stoichiometry
Source: mSystems. 2020 Jun 30;5(3):e00162-20. doi: 10.1128/mSystems.00162-20 (PMC7329320; doi:10.1128/mSystems.00162-20)
Supplement: TABLE S1 [file mSystems.00162-20-st001.docx]

| **Primer set (5’-3’)** | **Target**  **gene** | **Amplicon**  **length (bp)** | **Amplification efficiencies** | **Amplification**  **cycling conditions** | **References** |
| --- | --- | --- | --- | --- | --- |
|  |  |  |  |  |  |
| 338F (ACTCCTACGGGAGGCAGCAG)  518R (ATTACCGCGGCTGCTGG) | *16S rRNA* | 185 | 93-99% | 40 cycle (95 °C 30s,  60°C 5s, 72°C 34s) | 1 |
| ITS1 (TCCGTAGGTGAACCTGCGG) | *ITS* | 300 | 94-101% | 40 cycle (95 °C 30s, 60°C 5s, 72°C 34s) | 1 |
| 5.8s (CGCTGCGTTCTTCATCG) |  |  |  |  |  |
| CrenamoA23F (ATGGTCTGGCTWAGACG)  CrenamoA616R (GCCATCCATCTGTATGTCCA) | *amoA-a* | 624 | 98–102% | 40 cycle (95 °C 60s, 60°C 5s, 72°C 34s) | 2, 3 |
| Bac-amoA-1F (GGGGTTTCTACTGGTGGT) | *amoA-b* | 491 | 95–101% | 40 cycle (95 °C 60s, 60°C 5s, 72°C 31s) | 4, 5 |
| Bac-amoA-2R (CCCCTCKGSAAAGCCTTCTTC) |  |  |  |  |  |
| nifHF (AAAGGYGGWATCGGYAARTCCACCAC)  nifHRb (TGSGCYTTGTCYTCRCGGATBGGCAT) | *nifH* | 413 | 90–98% | 40 cycle (95 °C 60s, 60°C 5s, 72°C 34s) | 6, 7 |
| narG-f (TCGCCSATYCCGGCSATGTC) | *narG* | 110 | 101–105% | 40 cycle (95 °C 60s, 60°C 5s, 72°C 32s) | 8, 9 |
| narG-r (GAGTTGTACCAGTCRGCSGAYTCSG) |  |  |  |  |  |
| nirK876 (ATYGGCGGVCAYGGCGA)  nirK1040 (ATYGGCGGVCAYGGCGA) | *nirK* | 515 | 95–100% | 40 cycle (95 °C 60s, 60°C 5s, 72°C 34s) | 9, 10 |
| nirSCd3aF (AACGYSAAGGARACSGG) | *nirS* | 425 | 99–104% | 40 cycle (95 °C 60s, 60°C 5s, 72°C 34s) | 8, 10 |
| nirSR3cd (GASTTCGGRTGSGTCTTSAYGAA) |  |  |  |  |  |
| nosZ-F (CGCTGTTCITCGACAGYCAG)  nosZ-R (ATGTGCAKIGCRTGGCAGAA) | *nosZ* | 380 | 100–104% | 40 cycle (95 °C 60s, 60°C 5s, 72°C 34s) | 11, 12 |
| cnorB_B_F (AIGTGGTCGAGAAGTGGCTCTA) | *norB* | 372 | 93–97% | 40 cycle (95 °C 60s, 60°C 5s, 72°C 34s) | 12, 13 |
| cnorB_B_R (TCTGIACGGTGAAGATCACC) |  |  |  |  |  |
| ALPS-F730 (CAGTGGGACGACCACGAGGT)  ALPS-1101 (GAGGCCGATCGGCATGTCG) | *phoD* | 371 | 93–102% | 40 cycle (95 °C 30s, 60°C 5s, 72°C 34s) | 14, 15 |
| phoC-A-F1 (CGGCTCCTATCCGTCCGG)  phoC-A-R1 (CAACATCGCTTTGCCAGTG) | *phoC* | 155 | 96–105% | 40 cycle (95 °C 30s, 60 °C  5s, 72°C 34s) | 16, 17 |
| pqqCf1 (CATGGCATCGAGCAT GCT CC)  pqqCr1 (CAGGGCTGGGTCGCCAACC | *pqqC* | 312 | 92–97% | 40 cycle (95 °C 30 s, 58 °C  5s, 72°C 34s) | 18 |
| BPP-F (GACGCAGCCGA YGAYCCNGCNITNTGG) | *BPP* | 186 | 94–99% | 40 cycle (95 °C 60s, 57°C 30s, 72°C 45s) | 19, 20 |
| BPP-R (CAGGSCGCANRTCIACRTTRTT) |  |  |  |  |  |
| fungcbhIF (ACCAAYTGCTAYACIRGYAA) | *fungcbhIR* 100 98–102% 40 cycle (94 °C 30s, 48°C 45s, 72°C 90s) | | | | 21, 22 |
| fungcbhIR( GCYTCCCAIATRTCCATC) |  |  |  |  |  |
| GH31_350F (CAYCARTGYMGITGGGGNTA) | *GH31* 980 95–100% | | | 40 cycle (95 °C 45s, 50°C 45s, 72°C 100s) | 22 |
| GH31_660R (TTRTCICCNCCCCARTGNCC) |  |  |  |  |  |
| GH51_280F (AGNTGGCARTGGAAYGCNAC)  GH51_350R (ATYTGRTCDATIGCYTGYTG) | *GH51* 225 | | 96–104% | 40 cycle (95 °C 45s, 50°C 45s, 72°C 100s) | 22 |
| GH74_130F (TTYAARGTIGGIGGNAAYATG)  GH74_280R (CCRTCRTAIGGICCNGCNCC) | *GH51* 460 | | 98–105% | 40 cycle (95 °C 45s, 50°C 45s, 72°C 100s) | 22 |

*fungcbhIR*, fungal glycoside hydrolase family 7 cellobiohydrolase I genes; *GH31*, *GH51*, and *GH74*, glycoside hydrolase family 31, glycoside hydrolase family 51, and glycoside hydrolase family 74 genes, respectively; *amoA-a* and *amoA-b*, archaeal and bacterial ammonia monooxygenase genes, respectively; *BPP*, beta-propeller phytase.

**References**

1. Kong Y, Zhu C, Yang R, Luo G, Wang M, Ling N. 2018. Are the microbial communities involved in glucose assimilation in paddy soils treated with different fertilization regimes for three years similar? J Soils Sediment 1-15.
2. Könneke M, Bernhard AE, Jr DLT, Walker CB, Waterbury JB, Stahl DA. 2005. Isolation of an autotrophic ammonia-oxidizing marine archaeon. Nature 437:543-546.
3. Long X, Chen C, Xu Z, Oren R, He J. 2012. Abundance and community structure of ammonia-oxidizing bacteria and archaea in a temperate forest ecosystem under ten-years elevated CO_2_. Soil Biol. Biochem. 46:163-171.
4. Rasche F, Knapp D, Kaiser C, Koranda M, Kitzler B, Zechmeister-Boltenstern S. 2011. Seasonality and resource availability control bacterial and archaeal communities in soils of a temperate beech forest. The ISME J 5:89-402.
5. Szukics U, Hackl E, Zechmeister-Boltenstern S, Sessitsch A. 2012. Rapid and dissimilar response of ammonia oxidizing archaea and bacteria to nitrogen and water amendment in two temperate forest soils. Microbiol Res 167:103-109.
6. Yergeau E, Kang S, He Z, Zhou J, Kowalchuk GA. 2007. Functional microarray analysis of nitrogen and carbon cycling genes across an antarctic latitudinal transect. The ISME J. 1:163-179.
7. Morales SE, Cosart T, Holben WE. 2010. Bacterial gene abundances as indicators of greenhouse gas emission in soils. The ISME J 4:799-808.
8. Kandeler E, Brune T, Enowashi E, Dörr N, Guggenberger G, Lamersdorf N. 2009. Response of total and nitrate-dissimilating bacteria to reduced N input in a spruce forest soil profile. Fems Microbiol Ecol 67:444-454.
9. Bru D, Ramette R, Saby NPA, Dequiedt S, Ranjard L, Jolivet C. 2011. Determinants of the distribution of nitrogen-cycling microbial communities at the landscape scale. The ISME J 5:532-542.
10. Bárta J, Melichová T, Vaněk D, Picek T, Šantrůčková H. 2010. Effect of pH and dissolved organic matter on the abundance of nirK, and nirS, denitrifiers in spruce forest soils. Biogeochem 101:123-132.
11. Rich JJ, Heichen RS, Bottomley PJ, Cromack K, Myrold DD. 2003. Community composition and functioning of denitrifying bacteria from adjacent meadow and forest soils. Applied and Environ Microbio 69:5974-5982.
12. Luo G, Friman VP, Chen H, Liu M, Wang M, Guo S. 2018. Long-term fertilization regimes drive the abundance and composition of N-cycling-related prokaryotic groups via soil particle-size differentiation. Soil Biol Biochem 116:213-223.
13. Yu Y, Zhang J, Chen W, Zhong W, Zhu T, Cai Z. 2014. Effect of land use on the denitrification, abundance of denitrifiers, and total nitrogen gas production in the subtropical region of china. Biol Fert Soils 50:105-113.
14. Sakurai M, Wasaki J, Tomizawa Y, Shinano T, Osaki M. 2008. Analysis of bacterial communities on alkaline phosphatase genes in soil supplied with organic matter. Soil Sci Plant Nut 54:62-71.
15. Luo GW, Ling N, Nannipieri P, Chen H, Raza W, Wang M. 2017. Long-term fertilisation regimes affect the composition of the alkaline phosphomonoesterase encoding microbial community of a vertisol and its derivative soil fractions. Biol Fert Soil 53:375-388.
16. Gaiero JR, Bent E, Fraser TD, Condron LM, Dunfield KE. 2017. Validating novel oligonucleotide primers targeting three classes of bacterial non-specific acid phosphatase genes in grassland soils. Plant Soil 1-13.
17. Fraser TD, Lynch DH, Gaiero J, Khosla K, Dunfield KE. 2017. Quantification of bacterial non-specific acid (phoC) and alkaline (phoD) phosphatase genes in bulk and rhizosphere soil from organically managed soybean fields. Appl Soil Ecol 111:48-56.
18. Zheng BX, Hao XL, Ding K, Zhou GW, Chen QL, Zhang JB, Zhu YG. 2017. Long-term nitrogen fertilization decreased the abundance of inorganic phosphate solubilizing bacteria in an alkaline soil. Sci Rep 7:42284.
19. Huang H, Shi P, Wang Y, Luo H, Shao N, Wang G. 2009. Diversity of beta-propeller phytase genes in the intestinal contents of grass carp provides insight into the release of major phosphorus from phytate in nature. Appl Environ Microbiol 75:1508-1516.
20. Cotta SR, Cavalcante FDA, Seldin L, Andreote FD, Elsas JD. 2016. The diversity and abundance of phytase genes (β-propeller phytases) in bacterial communities of the maize rhizosphere. Lett Appl Microbiol 62:264-268.
21. Edwards IP, Upchurch RA, Zak DR. 2008. Isolation of fungal cellobiohydrolase I genes from sporocarps and forest soils by PCR. Appl Environ Microbiol 74:3481-3489.
22. Kellner H, Vandenbol M. 2010. Fungi unearthed: transcripts encoding lignocellulolytic and chitinolytic enzymes in forest soil. PLoS One 5:e10971.
